# Association of combined lead, cadmium, and mercury with systemic inflammation

**DOI:** 10.3389/fpubh.2024.1385500

**Published:** 2024-08-29

**Authors:** Emmanuel Obeng-Gyasi, Barnabas Obeng-Gyasi

**Affiliations:** ^1^Department of Built Environment, North Carolina A&T State University, Greensboro, NC, United States; ^2^Environmental Health and Disease Laboratory, North Carolina A&T State University, Greensboro, NC, United States; ^3^Indiana University School of Medine, Indianapolis, IN, United States

**Keywords:** environmental metals, systemic inflammation, C-reactive protein, Bayesian Kernel Machine Regression, NHANES, posterior inclusion probability

## Abstract

**Background:**

Exposure to environmental metals has been increasingly associated with systemic inflammation, which is implicated in the pathogenesis of various chronic diseases, including those with neurodegenerative aspects. However, the complexity of exposure and response relationships, particularly for mixtures of metals, has not been fully elucidated.

**Objective:**

This study aims to assess the individual and combined effects of lead, cadmium, and mercury exposure on systemic inflammation as measured by C-reactive protein (CRP) levels, using data from the National Health and Nutrition Examination Survey (NHANES) 2017-2018.

**Methods:**

We employed Bayesian Kernel Machine Regression (BKMR) to analyze the NHANES 2017-2018 data, allowing for the evaluation of non-linear exposure-response functions and interactions between metals. Posterior Inclusion Probabilities (PIP) were calculated to determine the significance of each metal's contribution to CRP levels.

**Results:**

The PIP results highlighted mercury's significant contribution to CRP levels (PIP = 1.000), followed by cadmium (PIP = 0.6456) and lead (PIP = 0.3528). Group PIP values confirmed the importance of considering the metals as a collective group in relation to CRP levels. Our BKMR analysis revealed non-linear relationships between metal exposures and CRP levels. Univariate analysis showed a flat relationship between lead and CRP, with cadmium having a positive relationship. Mercury exhibited a U-shaped association, indicating both low and high exposures as potential risk factors for increased inflammation. Bivariate analysis confirmed this relationship when contaminants were combined with lead and cadmium. Analysis of single-variable effects suggested that cadmium and lead are associated with higher values of the h function, a flexible function that takes multiple metals and combines them in a way that captures the complex and potentially nonlinear relationship between the metals and CRP. The overall exposure effect of all metals on CRP revealed that exposures below the 50th percentile exposure level are associated with an increase in CRP levels, while exposures above the 60th percentile are linked to a decrease in CRP levels.

**Conclusions:**

Our findings suggest that exposure to environmental metals, particularly mercury, is associated with systemic inflammation. These results highlight the need for public health strategies that address the cumulative effects of metal exposure and reinforce the importance of using advanced statistical methods to understand the health impact of environmental contaminants. Future research should focus on the mechanistic pathways of metal-induced inflammation and longitudinal studies to ascertain the long-term effects of these exposures.

## Introduction

Lead, cadmium, and mercury are pervasive environmental contaminants with a well-documented history of toxicity (1–4). Human exposure to these metals can occur through various routes, including inhalation, ingestion of contaminated food and water, and occupational exposure. Additionally, incidental ingestion of soils and household dust, are increasingly recognized as concerning exposure routes (5–7). Despite extensive regulation and efforts to reduce environmental contamination, these metals continue to pose a significant public health challenge due to their persistence in the environment and their potential for bioaccumulation in the human body (8, 9).

The association between metal exposure and systemic inflammation is biologically plausible, given the known mechanisms of metal-induced toxicity (10). These metals can induce oxidative stress by generating reactive oxygen species (ROS) (11, 12), which in turn can activate a range of inflammatory pathways. Additionally, these metals have been shown to disrupt the normal functioning of the immune system, either by directly affecting immune cells or by altering the expression of cytokines, chemokines, and other inflammatory mediators (13, 14).

This research article delves into the intricate relationship between these metals and systemic inflammation, a critical pathophysiological process underlying numerous chronic diseases. Systemic inflammation, characterized by the activation of immune pathways and the release of inflammatory mediators throughout the body, has been implicated in the progression of a variety of conditions, including cardiovascular diseases, neurodegenerative disorders, and certain cancers (15). When the body's immune system detects a threat, such as an infection, injury, or the presence of harmful substances like heavy metals, it triggers an inflammatory response to neutralize the threat and initiate healing processes. While acute inflammation is a protective mechanism, chronic systemic inflammation can become detrimental.

In cardiovascular diseases, systemic inflammation contributes to the development and progression of atherosclerosis, where inflammatory cells and mediators promote the formation of plaques in the arterial walls (16). This can lead to reduced blood flow, increasing the risk of heart attacks and strokes. Inflammatory markers like C-reactive protein (CRP) are often elevated in individuals with cardiovascular conditions, indicating ongoing inflammation that exacerbates these diseases.

Neurodegenerative disorders, such as Alzheimer's disease and Parkinson's disease, are also linked to systemic inflammation (17, 18). Chronic inflammation can lead to the activation of microglia, the immune cells of the brain, which release pro-inflammatory cytokines that damage neurons. This persistent inflammatory state contributes to the progressive loss of neuronal function and structure, leading to cognitive decline and motor impairments.

In the context of cancer, systemic inflammation creates a tumor-promoting environment. Inflammatory mediators can induce genetic mutations, promote tumor growth, and enhance the ability of cancer cells to invade surrounding tissues and metastasize to distant organs (19). Chronic inflammation is associated with increased cancer risk and poorer prognosis, as it supports the hallmarks of cancer, including sustained proliferative signaling, evasion of apoptosis, and angiogenesis.

Moreover, systemic inflammation is linked to metabolic disorders such as obesity and type 2 diabetes (20). Inflammatory cytokines interfere with insulin signaling, leading to insulin resistance, a key feature of type 2 diabetes. In obese individuals, adipose tissue becomes a source of chronic inflammation, contributing to the development of metabolic syndrome and associated complications.

In the context of assessing the impacts of environmental exposures, such as those from heavy metals like lead, cadmium, and mercury, on systemic inflammation, traditional analytical approaches often consider each pollutant in isolation (21). However, in real-world scenarios, individuals are typically exposed to a mixture of pollutants, rather than a single contaminant. This complexity necessitates the use of advanced statistical methods capable of evaluating the health effects of multiple pollutants simultaneously (22). One such method that has gained prominence in environmental health research is Bayesian Kernel Machine Regression (BKMR) (23).

BKMR is a novel statistical approach designed to address the challenges posed by multi-pollutant exposure analysis. This method allows researchers to evaluate the health effects of a mixture of pollutants, considering potential interactions and synergistic effects among the different components of the mixture (24). BKMR is particularly advantageous in its ability to handle highly correlated exposures and to provide insights into the combined and individual effects of each component in the mixture.

BKMR offers a comprehensive and nuanced approach in environmental health research, particularly for studying the effects of metal exposures like lead, cadmium, and mercury on systemic inflammation. This method allows for the evaluation of the collective impact of these metals, providing a holistic understanding of associated health risks (24, 25). The flexibility of BKMR in modeling non-linear relationships and varying sensitivities to different exposure levels is crucial, considering the complex nature of biological responses to toxicants (23, 26). Moreover, this approach not only assesses the joint effect of these metals on inflammation but also distinguishes the specific contribution of each individual metal, enhancing our understanding of their respective roles in systemic inflammation.

We chose to study lead, cadmium, and mercury due to their common co-existence in the environment, significant toxicological effects, and strong links to systemic inflammation. These metals are prevalent in industrial emissions, contaminated food and water, and certain consumer products, leading to higher combined exposure risks. Previous research has shown that they induce oxidative stress, a known pathway for systemic inflammation. Understanding their combined effects can inform risk assessment and the development of targeted strategies to mitigate exposure and protect public health.

## Materials and methods

### Study cohort and design

Data from the NHANES 2017-2018 was utilized in this investigation. This dataset is a representative sample of non-institutionalized people residing in all 50 U.S. states and the District of Columbia. The U.S. Centers for Disease Control and Prevention (CDC) collected the data, which are available in two-year cycles and include multi-year, stratified, multi-stage, and clustered samples. The NHANES employs a complex, multistage probability sampling design to ensure that the data is representative of the U.S. civilian non-institutionalized population. The 2017–2018 NHANES cycle includes thousands of participants, providing sufficient power to detect significant associations and enabling subgroup analyses. This large sample size enhances the robustness and credibility of the study results. Additionally, the data is collected by the CDC, ensuring rigorous data collection methods and high-quality standards. The consistency and reliability of the data make it a trusted source for epidemiological studies. The sampling process involves the selection of primary sampling units (PSUs), which are typically counties or groups of counties, followed by the selection of segments within PSUs, households within segments, and finally, individuals within households. Oversampling of certain subgroups, such as Hispanics, non-Hispanic Blacks, and low-income individuals, is conducted to improve the reliability and precision of health status indicator estimates for these groups. Selected individuals in the NHANES undergo a comprehensive physical examination conducted in mobile examination centers (MECs), which include detailed medical, dental, and physiological measurements. In addition to the physical examination, participants complete extensive interviews that collect demographic, socioeconomic, dietary, and health-related information. Blood samples are drawn from participants and sent to laboratories for the measurement of various biomarkers, including metal concentrations and C-reactive protein (CRP) levels. The NHANES dataset includes extensive quality control and quality assurance protocols to ensure the accuracy and reliability of the data. Data collection procedures are standardized, and staff are rigorously trained. The CDC continuously monitors data collection and laboratory procedures to maintain high standards of data quality. On the NHANES website of the CDC, additional descriptions and detailed information about the study design, sampling methodology, data collection procedures, and protocols are provided. Researchers can access comprehensive documentation and resources to understand and utilize the dataset effectively for their investigations (27).

### Metals and CRP measurements

#### Metals measurement

Metals in diluted whole blood were measured using inductively coupled plasma mass spectrometry (ICP-MS). ICP-MS is a validated technique widely recognized for its accuracy and precision in analyzing metals in biological media (28). All metal analytes in the dataset had the same detection limits. For analytes below the lower limit of detection, an imputed fill value was used, calculated as the lower limit of detection divided by the square root of 2. The NHANES Laboratory Procedures Manual provides detailed descriptions of specimen collection and processing. The National Center for Environmental Health (NCEH) within the CDC's Division of Laboratory Sciences performed the metal assays on whole blood samples using the ICP-MS method (Method No. ITB0001A).

#### CRP measurement

The concentration of C-reactive protein (CRP) in the blood was assessed using a two-reagent immunoturbidimetric approach. In this method, the blood sample was initially mixed with a Tris buffer and allowed to incubate. Following this, latex particles coated with mouse-derived antibodies against human CRP were added. These antibodies bind to CRP present in the sample, forming immune complexes that increase the solution's turbidity. This increase in turbidity, caused by light scattering, is proportional to the CRP concentration in the sample. The degree of light scattering was quantitatively measured at primary and secondary wavelengths of 546 nm and 800 nm, respectively. The resulting light absorbance was compared against a calibrated CRP standard curve to determine the CRP levels in the specimen.

These detailed and standardized procedures ensure the reliability and validity of the metal and CRP measurements in the NHANES dataset.

### Statistical analysis

Our study utilized linear regression and Bayesian Kernel Machine Regression (BKMR) analysis to evaluate the relationship between metal exposure and systemic inflammation. To ensure the integrity of our analysis, we addressed missing values in the variables of interest by imputing them with the median value. This approach helped to maintain a complete dataset and reduce potential bias associated with missing information.

Our data analytics approach began with thorough data cleaning to address any inconsistencies, duplicate records, or irrelevant information. This crucial step ensured that our analysis was based on accurate and reliable data. For any missing values within the variables of interest, we used median imputation, replacing missing values with the median value of the observed data. This method preserved the overall distribution of the data and minimized the impact of outliers.

We initially applied linear regression to examine the individual relationships between metal exposures and CRP levels, providing a preliminary understanding of potential associations. To capture the complex and potentially non-linear interactions between multiple metals and CRP levels, we then employed BKMR. This advanced statistical method allows for the evaluation of non-linear exposure-response functions and interactions between multiple exposures simultaneously, providing a more comprehensive analysis of the data.

These steps ensured a robust and thorough analytical process, enabling us to derive meaningful insights from the NHANES 2017-2018 data.

#### Descriptive statistics

Descriptive statistics are presented to describe the distribution of the exposure and demographic variables in the dataset and stratify them by the c-reactive protein. Spearman correlation was used to assess the relationships among the metal's exposure variables and c-reactive protein.

#### Bayesian Kernel Machine Regression (BKMR)

In this research, we employed Bayesian Kernel Machine Regression (BKMR) with the Markov Chain Monte Carlo (MCMC) sampling method, following the methodology outlined by Bobb et al. (24). Our process involved conducting 5,000 iterations to ensure robust analysis. The choice of priors in our BKMR model was guided by established Bayesian practices to ensure meaningful inference and computational efficiency. Specifically, we utilized non-informative priors for parameters where prior knowledge was limited, allowing the data to primarily inform the posterior distributions. Convergence diagnostics were meticulously conducted to validate the stability and reliability of our results. This included assessing trace plots, autocorrelation plots, density plots, and the Gelman-Rubin convergence statistics for each parameter, ensuring they exhibited stable and consistent patterns without trends. The Gelman-Rubin statistic was confirmed to be below 1.1, indicating successful convergence. A central component of our BKMR analysis was the use of Posterior Inclusion Probabilities (PIPs). PIPs, which range from 0 to 1, are critical in assessing the impact of individual metals within an environmental mixture. They help quantify the relative importance of each metal, such as lead, cadmium, and mercury, in influencing the outcome of interest. To understand the interaction between these metals and systemic inflammation, we computed high-dimensional exposure-response functions, denoted as h(z), at various intervals. This was done while keeping other influencing variables constant at their median values, allowing us to isolate the effects of each metal. BKMR's graphical interpretation capabilities were particularly valuable in our study. These features enabled us to visually compare the effects—both collective and individual—of metal exposures. Specifically, we could contrast outcomes observed at specific exposure percentiles against those at median exposure levels. This approach highlighted the unique relationships between each metal and the outcome while considering the constant median values of other exposures. We adjusted our analysis for potential confounders, including body mass index (BMI), gender, age, education, and ethnicity. This methodological approach provided a nuanced understanding of the individual and combined effects of metals like lead, cadmium, and mercury on systemic inflammation. The analyses were completed using R (version 4.2.3; R Foundation for Statistical Computing, Vienna, Austria)(29). The significance level was set at 0.05.

## Results

### Comparative analysis of critical study variables: CRP levels and their associations

The mean levels of critical study variables were explored by CRP levels above and below the median (Table 1). The results indicated that all variables apart from mercury had a statistically significantly higher level above the median for CRP as compared to below.

**Table 1:** Comparative analysis of study variables by median CRP levels: statistical significance and variations.

**Variable**	**CRP above median**	**CRP below median**	***p*-values**
Mercury (mean/SE)	1.04 (0.056)	1.15 (0.069)	0.134
Cadmium (mean/SE)	0.387 (0.017)	0.332 (0.010)	0.006
Lead (mean/SE)	1.01 (0.030)	0.928 (0.021)	0.026
BMI (mean/SE)	30.04 (0.288)	24.23 (0.178)	< 0.0001
Age (mean/SE)	40.98 (0.511)	34.89 (0.823)	< 0.0001

SE, Standard error.

**Figure 1:**
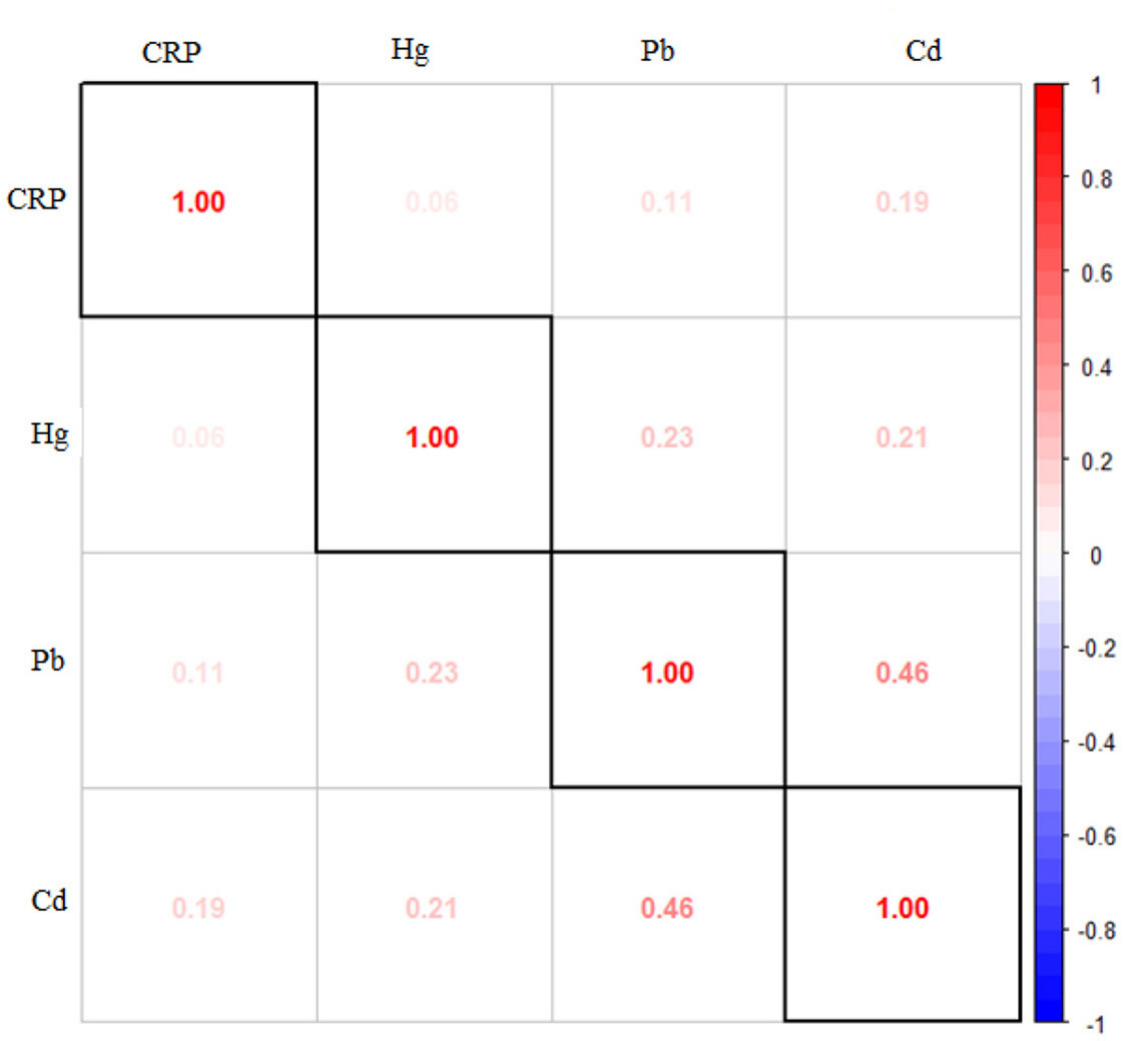
Spearman Correlation among variables of interest.

Figure 1 presents the Spearman correlation analysis conducted on the study's exposure and outcome variables. The results reveal strong correlations among the metals themselves, indicating inter-metal correlations. Other notable correlations are between CRP levels and cadmium, emphasizing the notable relationship between these two variables. Statistical analysis unveiled notable associations (*p* < 0.05) among various pairs of variables, including significant correlations between CRP and cadmium, lead and cadmium, lead and mercury, and cadmium and mercury.

### BKMR results

The significant correlations identified among the variables in our dataset signaled the necessity for employing Bayesian Kernel Machine Regression analysis as opposed to traditional linear regression methods. In traditional linear regression, the assumption of linearity between the independent and dependent variables is fundamental. However, in real-world scenarios like ours, where intricate and potentially nonlinear relationships exist among the variables, these linear methods may not capture the complexity of the data adequately.

BKMR, on the other hand, is a statistical technique that excels in situations where the relationships among variables are intricate and nonlinear. By utilizing flexible kernel functions and Bayesian modeling, BKMR helped to uncover hidden patterns, account for interactions, and capture intricate dependencies that linear regression models might overlook. This adaptability makes BKMR a powerful tool ultimately leading to more accurate and informative insights.

#### Quantifying metal-related factors in CRP variations: PIP and BKMR analysis

The Posterior Inclusion Probability (PIP) for each metal concerning its relationship with CRP serves as a metric quantifying the likelihood of each contaminant playing a significant role in explaining the variations observed in CRP levels. The PIP values for the influence of lead, cadmium, and mercury on systemic inflammation are 0.3528, 0.6456, and 1.000, respectively.

Table 2 provides hierarchical BKMR analysis for CRP. The analysis categorizes exposure variables into a group and presents the Group PIP and Conditional PIP (values for the group). For CRP, group 1 includes metals (lead, cadmium, and mercury) all of which have group PIP values of 1 but only mercury has a high condition PIP of 0.9868 suggesting a major influence on CRP.

**Table 2:** BKMR analysis of systemic inflammation: group and conditional posterior inclusion probabilities for lead, cadmium, and mercury.

**Variable**	**Group PIP**	**Conditional PIP**
Lead	1	0.01000
Cadmium	1	0.0032
Mercury	1	0.9868

#### Univariate analysis: examining the isolated effects of mercury, cadmium, and lead on CRP

**Figure 2:**
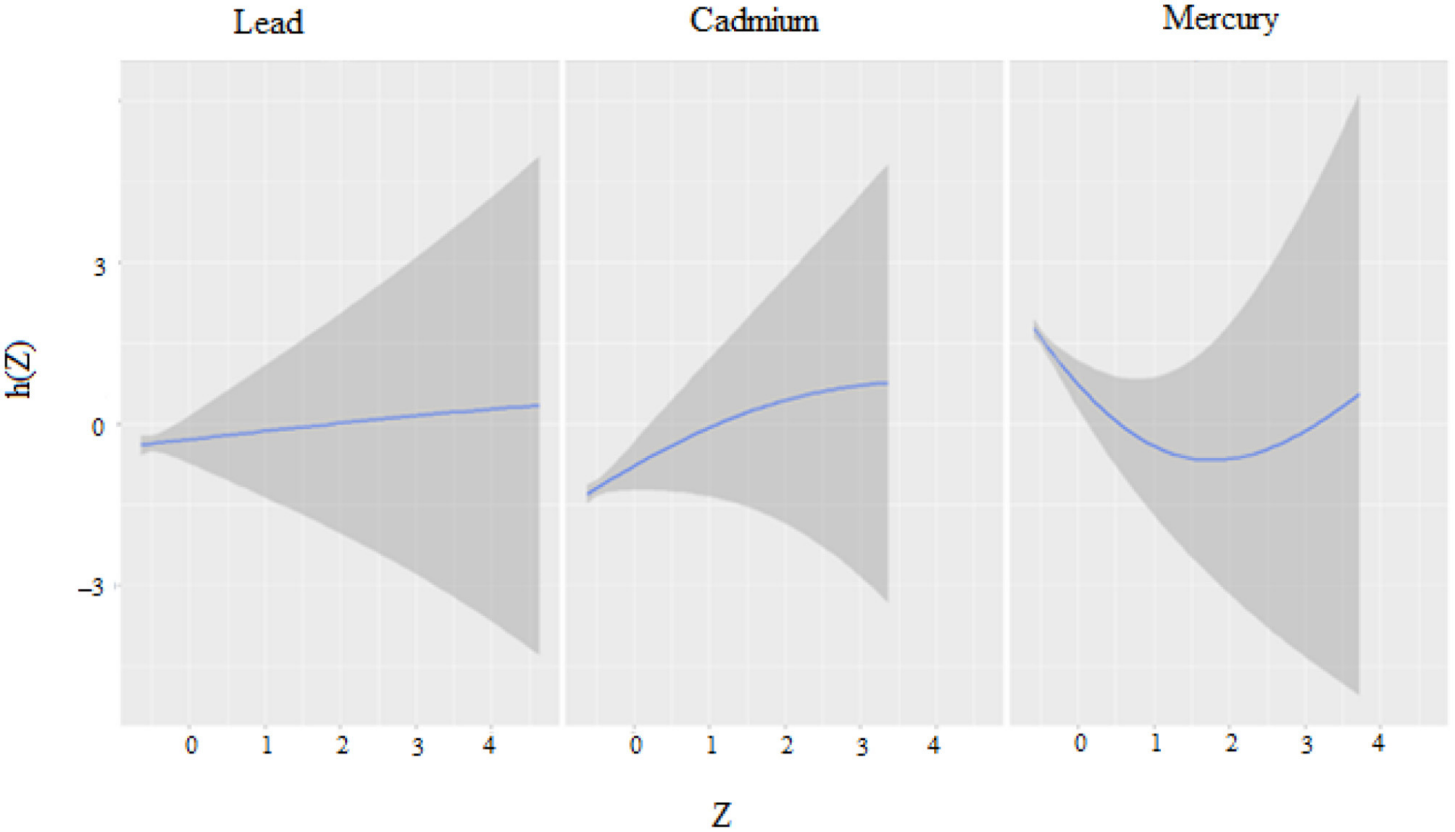
Univariate exposure–response functions and 95% credible interval for association between single metal exposure when other metals exposures are fixed at the median.

The univariate approach visually examines the individual effect of Mercury, Cadmium, and Lead on CRP. Figure 2 shows the impact of each metal on CRP when the other metals are fixed at the median and the covariates are held constant with cadmium and mercury having the largest impact. **Regarding the figure**, the flat curve in the Lead panel suggests that variations in Lead exposure do not significantly affect CRP levels across the range of exposures analyzed. This could mean that Lead, within the study's observed exposure range, might not be a major determinant of CRP levels, or that its effect is overshadowed by other factors not captured in this plot.

The curve for Cadmium rises at lower exposure levels before plateauing, indicating that an increase in Cadmium exposure may be associated with higher CRP levels initially. However, as exposure continues to increase, this effect does not appear to intensify. This might suggest a threshold effect, where below a certain level of exposure, changes in Cadmium concentrations have a more pronounced impact on CRP levels.

The U-shaped curve observed for Mercury implies a non-linear relationship with CRP levels. Low and high levels of Mercury exposure are associated with higher CRP levels, whereas moderate levels correlate with lower CRP. This could be indicative of a complex mechanism by which Mercury affects inflammation, potentially having a hormetic effect where it might exert different biological effects at different concentrations.

### Visualizing bivariate exposure-response functions with fixed percentile values

**Figure 3:**
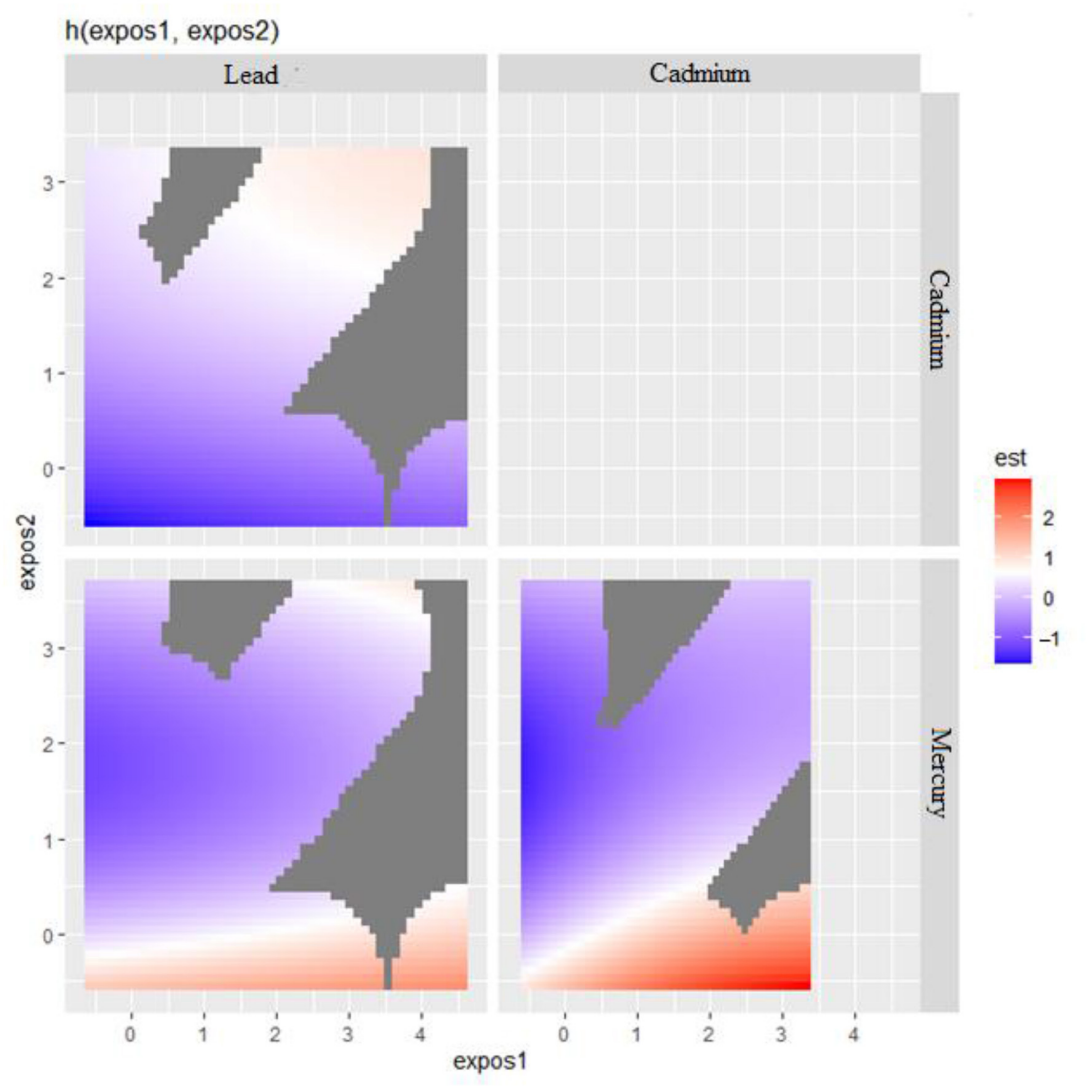
Bivariate exposure-response function of metals with CRP.

Bivariate metals exposure on CRP was explored where two metals of interest effects on CRP were examined while all other predictors were fixed at a particular percentile. In these plots (Figure 3), the color scale (est) represents the estimated effect on the health outcome. In this plot, red indicates a higher positive effect (which means an increased risk of a negative health outcome associated with increasing levels of the biomarker CRP), blue indicates a negative effect, and white or gray indicates no effect. The results seen in Figure 3 suggest that in the ‘Lead' vs. ‘Cadmium' plot (top left), higher levels of both exposures seem to have no effect on the outcome as indicated by the white and blue regions. In the ‘Cadmium' vs. ‘Mercury' plot (bottom right), there appears to be a region where increasing levels of both ‘Cadmium' and ‘Mercury' are associated with a positive effect on the outcome, as indicated by the red area. This happens also with Lead and Mercury but to a lesser extent.

**Figure 4:**
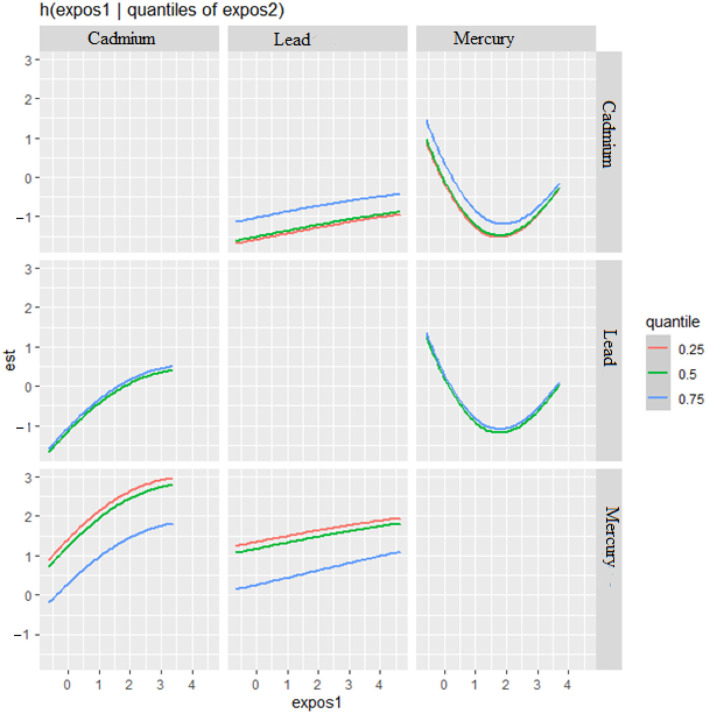
Bivariate exposure-response function of metals with CRP–investigating predictor-response function with varying quantiles of the second predictor, while other predictors are fixed.

The bivariate relationship was further explored by examining metal pairs. The analysis examined the relationship between individual metals and CRP by fixing the second metal at different quantiles: 25th (red line), 50th (green line), and 75th (blue line), with other metals held at the median (Figure 4). These models were adjusted for the covariates of interest. The x-axis, labeled “expos1”, shows the levels of one exposure, while the y-axis, labeled “est”, represents the estimated effect on CRP levels. Each row of plots corresponds to a different exposure being considered as “expos1”.

**Interaction Effects**: Each plot shows how the relationship between “expos1” and CRP changes at different quantiles of a second exposure, “expos2”. The three lines within each plot correspond to the 25th, 50th, and 75th quantiles of “expos2”, as indicated by the color legend.

The interpretation of the plot by each metal is as follows. **Cadmium (as expos1)**: When interacting with Cadmium (top row), the effects on CRP appear relatively flat across all quantiles of Lead and Mercury, suggesting that Cadmium's effect on CRP levels is consistent regardless of the levels of the other metals.

**Lead (as expos1)**: For Lead, the plots show a U-shaped relationship with CRP at different quantiles of Cadmium and Mercury, indicating that both low and high levels of Lead are associated with higher CRP levels, suggesting a non-linear interaction.

**Mercury (as expos1)**: Mercury's interaction plots show a strong U-shaped relationship with CRP at different quantiles of Cadmium and a similar but less pronounced U-shape with Lead. This suggests that Mercury has a non-linear association with CRP levels, potentially indicating a more complex interaction.

**Effect of Quantiles**: The differences in the shapes of the lines across different quantiles of “expos2” within each plot indicate how the effect of “expos1” on CRP varies with the levels of “expos2”. For example, in the bottom left plot (Mercury interacting with Cadmium), the curves for the 25th and 50th quantiles of Cadmium are relatively similar, suggesting consistent effects at lower to mid-levels of Cadmium. However, at the 75th quantile, the curve rises more steeply, suggesting a stronger interaction effect of Mercury on CRP at higher levels of Cadmium.

### Overall risk summary of CRP levels in relation to exposure percentiles

**Figure 5:**
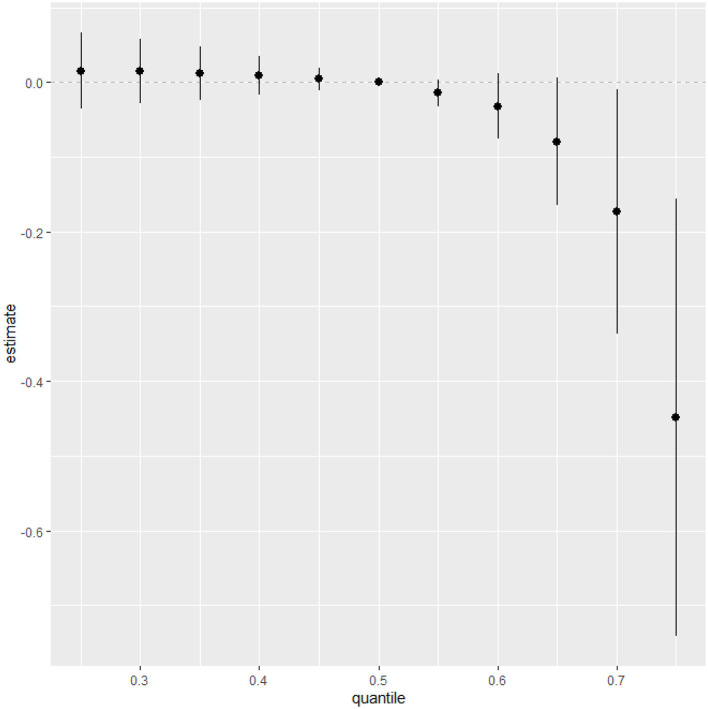
Summary of overall health effects of the exposures (multimixers) on the outcome depends on various percentiles (form 25th to 75th percentiles).

Figure 5 measures the total effect of all exposures or mixtures. The exposures are fixed at different quantities starting from the 25th percentile to 75th percentile at increments of 5 using the 50th percentile (median) to compare the exposures. The estimation for all exposures at the 50th percentile shown at zero (dashed line) demonstrates that when comparing all exposures between the 20th and 55th percentile exposure level to the 50th percentile exposure level, the CRP is above zero while after the 60th percentile exposure level CRP falls below zero. This analysis reveals that exposures below the 50th percentile exposure level are associated with an increase in CRP levels, while exposures above the 60th percentile are linked to a decrease in CRP levels.

### Single-variable effects of metals on CRP

**Figure 6:**
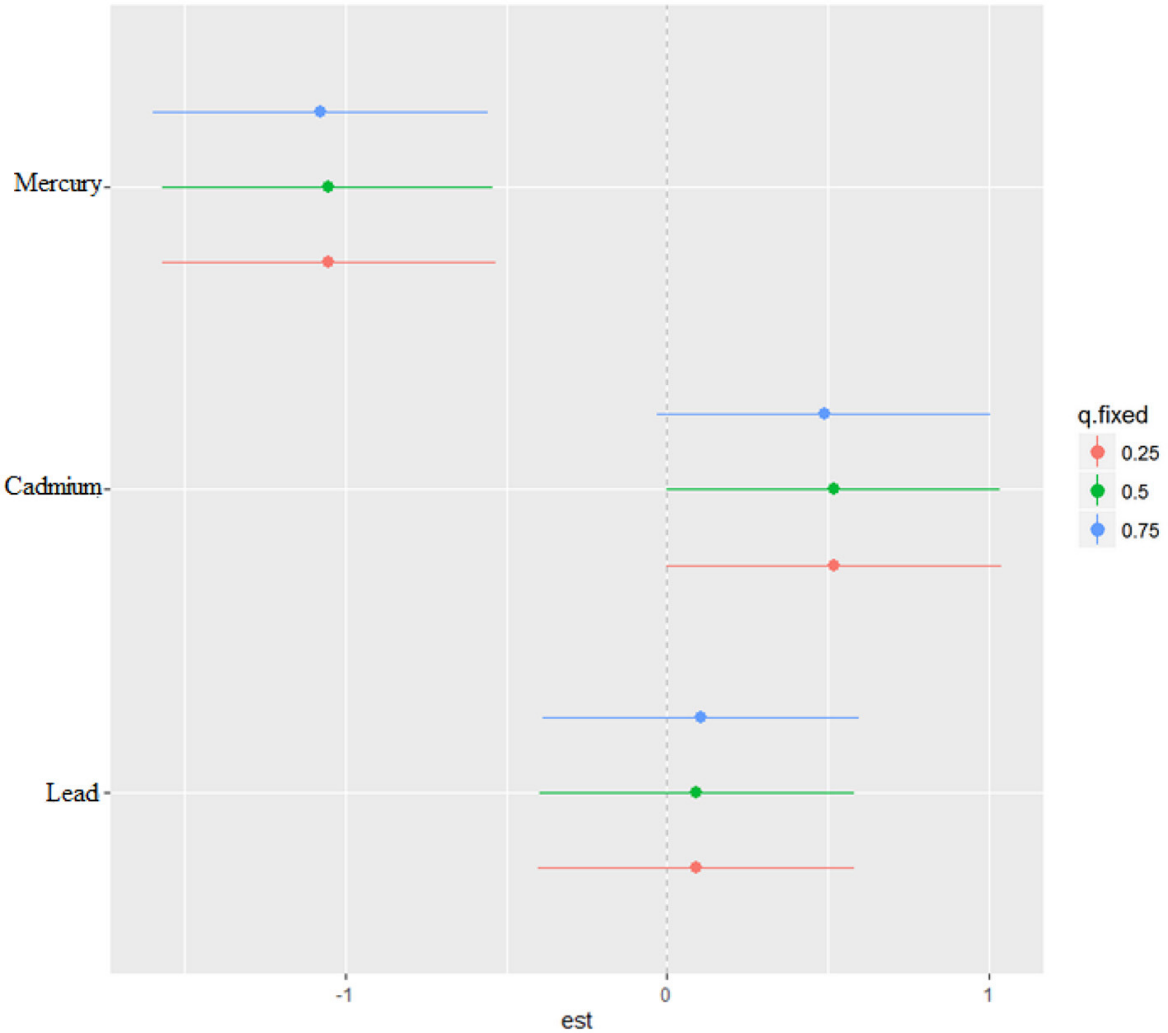
Single-variable effect of metals at increasing quartiles for CRP.

The single-variable effect helps to understand the effect of a single predictor at different quantiles giving us the ability to assess their contribution to the overall risk of elevated CRP. Figure 6 demonstrates the single-variable effects of metals on CRP at the 75th (blue), 50th (green), and 25th (red) quantile and suggest cadmium and lead are associated with higher values of the ***h*** function, a flexible function which takes multiple metals and combines them in a way that captures the complex and potentially nonlinear relationship between the metals and CRP. Overall, the plot and the quantiles specifically show how the relationship between each metal and CRP may change across the distribution of the metal exposure.

## Discussion

This study embarked on a nuanced exploration of the relationships between exposure to various heavy metals and systemic inflammation, as quantified by C-reactive protein levels, utilizing the robust Bayesian Kernel Machine Regression (BKMR) methodology. Our results substantiate the proposed link between metal-induced oxidative stress and heightened systemic inflammation, drawing attention to the intricate interplay between lead, cadmium, and mercury and their collective influence on CRP. Studying the combined effects of heavy metals like lead, cadmium, and mercury provides a more accurate assessment of health risks. These metals often coexist in the environment, and their interactions can amplify their harmful effects on systemic inflammation and overall health. Understanding the combined impact of these metals can inform more effective public health policies and regulations. Policies can be tailored to address the cumulative risks posed by mixed metal exposures, leading to better protection for at-risk populations. Highlighting the combined effects of heavy metals is crucial for promoting environmental and health justice. Marginalized communities often face higher exposures to multiple pollutants, and this research can support the development of strategies to reduce environmental health disparities and ensure equitable health outcomes for all communities. By recognizing the complex interactions between different heavy metals, healthcare providers can develop more effective interventions and treatments. This knowledge can lead to better screening protocols, preventive measures, and treatment plans for individuals exposed to these harmful substances. Studying the combinations of heavy metals advances scientific understanding of their synergistic effects. This knowledge is essential for developing innovative solutions to mitigate the health impacts of environmental contaminants and improve public health outcomes.

Through the lens of BKMR, we were able to unveil complex non-linear relationships and potential synergistic interactions among metal exposures, phenomena that remain obscured within the confines of conventional linear models. Mercury, in particular, emerged with a pronounced non-linear relationship with CRP levels, a biphasic pattern suggesting that both deficient and excessive exposures bear the potential to exacerbate inflammation. The complexity of Mercury's relationship with health outcomes has been demonstrated in other studies (30). Mercury's effects on inflammation are well known (31), but these findings add to the nuance of how exposure context shapes inflammation-related outcomes. This nuanced understanding has profound clinical implications, especially for populations burdened with high environmental exposure, prompting a shift in public health initiatives to consider the intricate and cumulative effects of metal exposures.

To further dissect these complexities, we leveraged the Posterior Inclusion Probability (PIP) as an analytical compass to gauge the significance of each metal's role in the observed variations in CRP levels. Mercury's unequivocal PIP of 1.000 firmly establishes its significant influence on CRP levels, indicating its strong role in systemic inflammation. Cadmium's substantial, albeit less consistent PIP of 0.6456, along with lead's more modest PIP of 0.3528, paint a more heterogeneous picture of influence, suggesting that their impact on inflammation may be modulated by a confluence of exposure levels (32), biological interactions (33), and other methodological nuances of the model. The substantial impact of cadmium in a mixture have been noted elsewhere (34).

The collective importance of these metals is underscored by Group PIP values of 1, yet it is mercury, with a high conditional PIP of 0.9868, that stands out as a pivotal individual factor in the elevation of CRP levels. This differentiation in the PIP spectrum not only holds clinical weight but also kindles a policy discourse on prioritizing interventions (35) to curtail exposures, with a particular focus on mercury.

Our comparative analysis across critical study variables disclosed a notable divergence, with cadmium and lead exposures correlating with statistically higher CRP levels, an affirmation of their differential impact on inflammation markers. While mercury did not exhibit a similar direct correlation, its U-shaped response curve in the BKMR analysis reveals a potential hormetic effect (36), signifying that varying exposure levels may instigate distinct biological responses. The U-shape may also be due to their mechanism. Specifically, at low levels, mercury exposure might stimulate inflammatory pathways or immune responses, potentially through the activation of oxidative stress or inflammatory signaling pathways. Conversely, at high levels, mercury's toxic effects could overwhelm these pathways, leading to immunosuppression or reduced inflammation. This dual effect could explain the observed U-shaped curve. Additionally, Previous studies have reported similar U-shaped dose-response relationships for other toxicants, suggesting that the effect of mercury on inflammation may not be linear (30). For instance, some research has shown that low-level exposure to certain metals can enhance pro-inflammatory cytokine production (37), while high levels may induce apoptosis or other protective mechanisms that reduce inflammation (38).

Cadmium's threshold effect, with a plateauing of CRP levels in response to increasing exposure, suggests a saturation point in its inflammatory potential, whereas the absence of a pronounced dose-response relationship for lead signals a more intricate or subdued influence on inflammation.

The outcome of the bivariate exposure-response functions further illuminated the potential for synergistic interactions between metals (39), particularly in the dynamic interplay between lead and cadmium and between cadmium and mercury. This synergy, which could amplify inflammation, underscores the need for public health policies (40) to address the multifaceted risk of mixed metal exposures.

Our analysis also highlighted a paradoxical inverse relationship at higher metal exposure percentiles, where increased metal levels were correlated with a decrease in CRP, hinting at possible saturation effects or adaptive biological mechanisms that mitigate inflammation at heightened concentrations of these metals.

The implications of our findings are manifold, extending beyond immediate clinical concerns to inform future research agendas. For example, in the context of our findings on metal-induced oxidative stress and inflammation, the role of CRP as a clinical biomarker gains additional significance. CRP, produced in response to inflammation, serves as a crucial indicator for a range of conditions, including those within the neurosurgical sphere. Elevated CRP levels, linked to an increased risk of neurodegenerative diseases and stroke, underscore the broader implications of metal exposure in systemic inflammation (41). This insight is vital in, for example, neurosurgery, where understanding such inflammatory markers can profoundly impact surgical outcomes and recovery processes (42). Thus, our study's revelation of the nuanced influence of metals like mercury on CRP levels brings to light the critical intersection of environmental health and neurosurgical care.

The need for advanced statistical tools to decipher the labyrinth of complex environmental exposures is clear. Future research should focus on elucidating the mechanistic pathways by which these metals influence inflammation and the progression of related chronic diseases. Longitudinal studies are particularly warranted to unravel the temporal intricacies between metal exposure and inflammation, potentially paving the way for targeted therapeutic and preventive measures. Moreover, public health policies must adapt to address the complex and cumulative risks posed by mixed metal exposures, emphasizing the need for stricter regulations and interventions, particularly concerning mercury. Clinicians and policymakers should collaborate to develop strategies that mitigate metal exposure, enhance environmental safety, and improve health outcomes for affected populations.

One limitation of our study is the lack of specific geographical information in the NHANES dataset, as it is de-identified to protect participant privacy, which precludes analysis of localized environmental exposure risks. Another limitation of our study is that we focused on the end result of systemic inflammation rather than incorporating parameters such as morbid obesity, waist circumference, and other well-known contributors to systemic inflammation and elevated CRP levels, which could provide additional context and enhance the understanding of the relative impact of heavy metal exposures. Additionally, the cross-sectional design of the NHANES dataset limits our ability to infer temporality and causality between heavy metal exposures and systemic inflammation. Longitudinal studies are needed to better understand the temporal relationships and causal pathways involved.

## Conclusions

This study highlights the complex interactions between lead, cadmium, and mercury exposures and systemic inflammation, as measured by C-reactive protein (CRP) levels. Utilizing Bayesian Kernel Machine Regression (BKMR) and Posterior Inclusion Probabilities (PIPs), we revealed significant non-linear relationships, particularly noting mercury's pronounced U-shaped association with CRP. The findings underscore the importance of considering combined metal exposures in public health strategies. Future research should focus on the mechanistic pathways and long-term effects of these exposures to better inform policy and therapeutic interventions.

## Data Availability

Publicly available datasets were analyzed in this study. This data can be found here: cdc.gov/nchs/nhanes/index.htm.
